# The beauty of a quadricuspid aortic valve from the multimodality perspective of the Heart Team

**DOI:** 10.1093/ehjcr/ytae684

**Published:** 2025-01-13

**Authors:** Alexandru Patrascu, Hossam Homedan, Ilka Ott

**Affiliations:** Department of Cardiology, Rhythmology, Electrophysiology and Angiology, Helios Hospital Pforzheim, Kanzlerstrasse 2-6, 75175 Pforzheim, Germany; Helios Hospital Karlsruhe, Department for Cardiac Surgery, Franz-Lust-Strasse 30, 76185 Karlsruhe, , Germany; Department of Cardiology, Rhythmology, Electrophysiology and Angiology, Helios Hospital Pforzheim, Kanzlerstrasse 2-6, 75175 Pforzheim, Germany

## Case description

**Figure 1:**
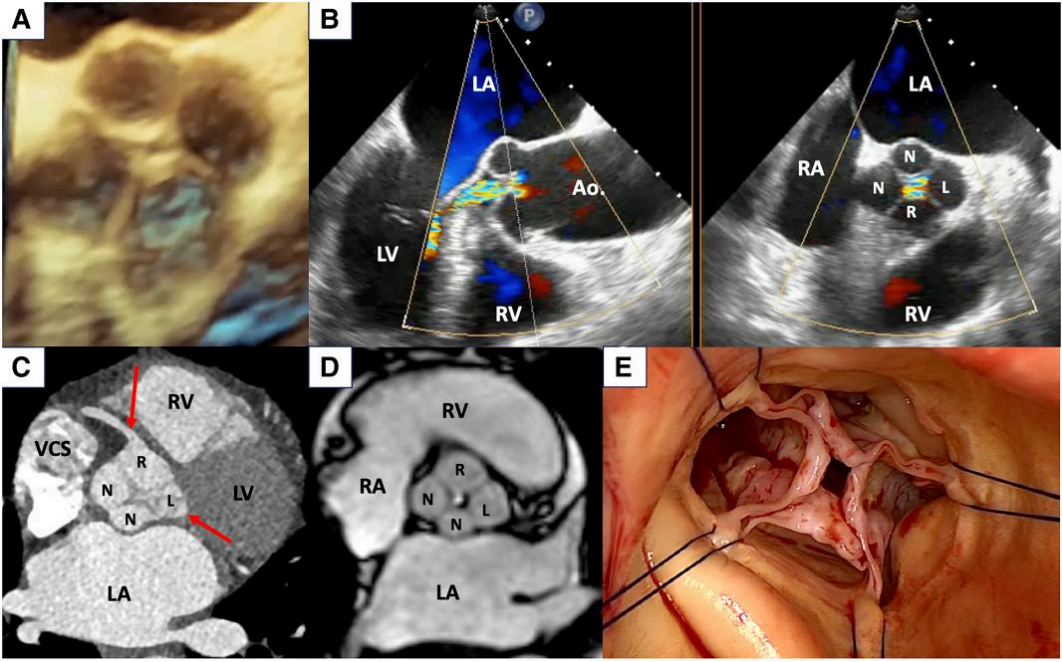
Multimodality imaging of a quadricuspid aortic valve with high-grade regurgitation, as assessed by three-dimensional transesophageal echocardiography (*A*), biplane midesophageal transesophageal echocardiography (*B*), cardiac computed tomography (*C*), cardiac magnetic resonance imaging (*D*), and surgical views (*E*). The ostia of the two coronary arteries are marked by red arrows (*C*). N, non-coronary; R, right coronary; L, left coronary; RA, right atrium; LA, left atrium; RV, right ventricle; LV, left ventricle; VCS, superior vena cava; Ao., ascending aorta.

A 62-year-old female with shortness of breath (New York Heart Association Class III) for several months was referred for transesophageal echocardiography (TOE) after higher degree aortic valve regurgitation (AR) was detected by outpatient transthoracic echocardiography. Transesophageal echocardiography revealed an extremely rare quadricuspid aortic valve (*Figure 1A*, Supplementary material online, *Video S1*) with two non-coronary cusps (N, non-coronary), preserved orifice area, and central coaptation gap (*Figure 1B*; RA, right atrium; LA, left atrium; RV, right ventricle; LV, left ventricle; Ao., ascending aorta). This resulted in moderate-to-severe AR, as measured by vena contracta of 8 mm, pressure half-time of 250 ms, effective regurgitant orifice area of 0.28 cm^2^, and diastolic flow reversal in the descending aorta. Cardiac computed tomography confirmed the rare anatomy (*Figure 1C*, Supplementary material online, *Video S2*; SVC, superior vena cava), and showed normal origin of both coronary arteries (red arrows), from their respective cusps (R, right coronary, L, left coronary). No coronary stenoses were present. On top, cardiac magnetic resonance imaging ruled out other congenital anomalies, and calculated an aortic regurgitation fraction of 35% (*Figure 1D*, Supplementary material online, *Video S3*). The left ventricle (LV) had preserved ejection fraction (62%) but showed relevant dilatation (end-systolic diameter 47 mm, end-diastolic volume index 101 mL/m^2^). The case was discussed on an interdisciplinary basis by cardiologists and cardiac surgeons. In view of the symptoms, increased NTproBNP (982 pg/mL), and LV enlargement, the ‘Heart Team’ decided on surgical treatment. Perioperative assessment confirmed a Type A valve^1^ with four relatively equal cusps, which, in theory, makes reconstruction difficult.^2^ Nonetheless, tricuspidization was attempted, but proved to be ineffective, so the aortic valve (*Figure 1E*, Supplementary material online, *Video S4*) was replaced without complications by a biological prosthesis via partial sternotomy. The in-hospital stay was uneventful. The patient was transferred to a rehabilitation clinic on post-operative Day 7, and has been symptom-free ever since. This case highlights the diagnostic approach in evaluation of quadricuspid aortic valve with severe AR.

## Supplementary Material

ytae684_Supplementary_Data

## Data Availability

The data underlying this article will be shared on reasonable request to the corresponding author.
